# Topography of generalized periodic epileptiform discharges in postanoxic nonconvulsive status epilepticus

**DOI:** 10.1002/epi4.12073

**Published:** 2017-08-21

**Authors:** Dimitris Fotis Sakellariou, George Kostantinos Kostopoulos, Mark Philip Richardson, Michalis Koutroumanidis

**Affiliations:** ^1^ Department of Clinical Neurophysiology and Epilepsy Guy's and St. Thomas’ National Health Service Foundation Trust London United Kingdom; ^2^ Division of Neuroscience Institute of Psychiatry, Psychology, and Neuroscience King's College London London United Kingdom; ^3^ Neurophysiology Unit Department of Physiology School of Medicine University of Patras Patras Greece

**Keywords:** Hypoxic coma, Cardiac arrest, Voltage maps, EEG, Intensive care

## Abstract

We studied slow (≤2.5 Hz) nonevolving generalized periodic epileptiform discharges (GPEDs) in the electroencephalogram (EEG) of comatose patients after cardiac arrest (CA) in search of evidence that could assist early diagnosis of possible hypoxic nonconvulsive status epilepticus (NCSE) and its differentiation from terminal brain anoxia (BA), which can present with a similar EEG pattern. We investigated the topography of the GPEDs in the first post‐CA EEGs of 13 patients, using voltage‐mapping, and compared findings between two patients with NCSE and GPEDs > 2.5 Hz (group 1), and 11 with GPEDs ≤ 2 Hz, of whom six had possible NCSE (group 2) and five had terminal BA (group 3). Voltage mapping showed frontal maximum for the negative phase of the GPEDs in all patients of groups 1 and 2, but not in any of the patients of group 3, who invariably showed maximization of the negative phase posteriorly. Morphology, amplitude, and duration of the GPEDs varied across the groups, without distinctive features for possible NCSE. These findings provide evidence that, in hypoxic coma after CA with slow GPEDs, anterior topography of the maximum GPED negativity on voltage mapping may be a distinctive biomarker for possible NCSE contributing to the coma.

In postanoxic coma after cardiac arrest (CA), the electroencephalogram (EEG) is indispensable for the evaluation of the severity of the brain injury and the early diagnosis of nonconvulsive status epilepticus (NCSE). Hypoxic NCSE that may significantly contribute to the coma, but also severe and irreversible, near‐terminal brain anoxia (BA), are commonly associated with nonevolving generalized periodic epileptiform discharges (GPEDs), defined as bilateral, synchronous, and symmetric spikes or spike–wave complexes with relative uniform morphology and duration, a quantifiable interdischarge interval between consecutive waveforms, and recurrence of the waveform at nearly regular intervals.^1^ Recognition of NCSE primarily relies on GPED frequency, assessed by visual inspection. Whereas a diagnosis of NCSE can be made with frequencies > 2.5 Hz, an additional criterion is required for slower frequencies, namely both EEG and clinical improvement after intravenous (IV) administration of a rapidly acting antiepileptic drug (AED). EEG improvement alone only suggests possible NCSE.^2^ By implication, slow GPEDs that fail to meet the criteria of possible NCSE are considered an epiphenomenon of BA. Although the postanoxic occurrence of GPEDs carries poor prognosis,^3^ and some authors believe that aggressive treatment may not be warranted in such patients,^4^ AED treatment has been associated with grade 3 cerebral performance outcome grade in some patients with possible NCSE.^5^

In clinical practice, slow (≤2.5 Hz) GPEDs present a particularly difficult diagnostic challenge. Some patients with EEG but not clinical improvement after IV AED may be diagnosed with possible NCSE for reasons that may include heavy pharmacological suppression, coexisting sepsis or metabolic derangements, a degree of secondary (potentially reversible) cerebral edema, comorbidities like dementia, or simply insufficient noxious stimulation. Some of these factors may also account for the abnormal EEG background sometimes seen after a partial EEG response. Moreover, a diagnosis of possible NCSE may be missed when the dose of IV AED is insufficient to block GPEDs, or when GPEDs are not responsive to benzodiazepines but only to temporary propofol withdrawal, which may not be routinely attempted.^6^ Very low (i.e., <2 Hz) frequencies may sometimes discourage vigorous diagnostic maneuvers.

Efforts to bypass such constraints have focused on the GPED morphology, but discrimination between “epileptic” (associated with NCSE) and “nonepileptic” (associated with BA) GPEDs appears impossible at the individual patient level.^6^, ^7^ We used event‐related computational methods to study “slow” GPED microstructure, aiming to detect possible characteristic features that could assist early differentiation between possible NCSE presumably contributing to coma and BA.

## Methods

### Patient groups

We investigated GPEDs in three groups of comatose post‐CA patients (Table 1). Group 1 includes two patients, diagnosed with NCSE on the basis of >2.5 Hz GPEDs in the first postinsult video‐EEG (2/2), EEG (1/1) and clear clinical response (1/1) to IV AED, and final resolution of the NCSE with normalization or near‐normalization of the biological EEG rhythms and clinical improvement. All patients in groups 2 and 3 had ≤2.5‐Hz GPEDs on their first EEG. In group 2 (six patients), the diagnosis of possible NCSE contributing to coma was based on clear EEG (but not clinical) responses to acute IV AED/temporary propofol withdrawal (five of five; see also Fig. S1), or aggressive escalation of IV AED (1/1). In the five patients of group 3, GPEDs were deemed to reflect terminal BA, because none showed any EEG or clinical improvement to IV AED/propofol maneuver. There was no difference in terms of comorbidities or AED treatment between the patients of groups 2 and 3; all received IV levetiracetam, phenytoin, or valproate acid in comparable doses. All patients underwent 24‐h therapeutic hypothermia or active normothermia (multicenter Target Temperature Management trial,^8^ ethics approval registered at ClinicalTrials.gov NCT01020916).

### EEG recordings

Computational analysis was performed on the first postinsult EEGs, performed in the intensive care unit of St. Thomas’ Hospital, London, United Kingdom (EEG methodology is provided in the Supplementary Material).

### Event selection and analysis

All patients had GPEDs that were nonevolving in frequency, morphology, or location.^1^, ^2^ GPED frequencies were nearly regular in all EEGs, with maximum fluctuations not exceeding 0.8 Hz (Table 1).

For each patient, all artifact‐free GPED events were visually annotated at the peak of the first maximal in amplitude negative phase using precise time markers. A minimum period of 5 seconds between marked events was left without annotations to avoid overlap in the event‐related averaging analysis. The morphology of the GPED waves was estimated via time averaging in the electrodes where the GPEDs were more prominent according to the calculated voltage maps.

Event‐related data were further processed by a custom‐made MATLAB‐based (MathWorks, Natick, MA, U.S.A.) software suite developed at the Neurophysiology Unit, School of Medicine, University of Patras, Greece. Scalp voltage mapping of the annotated events was performed by the use of multiquadric interpolation based on Green's function method^9^ after baseline correction of all channels. Additionally, the annotated events were time‐averaged and superpositioned within a time window of −0.50 to 0.50 s, centered at time = 0.00.

**Table 1:** Clinical and EEG features of patients

	Patients/sex/age	Reactivity			Outcome^a^	GPEDs frequency (Hz)	GPEDs Amplitude (uV)
		Anti‐seizure manoeuver	EEG response	Clinical Response			
Group 1							
Patient 1	1 / F / 52	None	N/A	N/A	3	2.5–3.0	−275.6
Patient 2	2 / M / 55	DZP	GPEDs ceased; θ, α and β	Opened eyes to command	2	2.5–3.0	−187
Group 2							
Patient 3	3 / M / 63	LRZ	GPEDs ceased; δ, θ, α and β	No	3	1.5–2	−55.69
Patient 4	4 / M / 62	CNZ	GPEDs ceased; δ and θ	No	3	0.8–1.5	−34.8
Patient 5	5 / M / 60	Propofol window	GPEDs ceased; δ and θ, some α	No	5	1	−110.7
Patient 6	6 / M / 60	None	N/A^b^	No response	3	1.2–2.0	−83
Patient 7	7 / M / 71	DZP	GPEDs ceased; δ and θ, some α	No	3	1.2–1.8	−130.6
Patient 8	8 / M / 70	Propofol window	No physiological rhythms, but focal frontal seizure activity	No	5	0.8–1.2	−74.8
Group 3							
Patient 9	8 / M / 58	LRZ	No Effect	No response	3	1.5–2.0	−86.4
Patient 10	9 / M / 78	LRZ	No Effect	No response	5	0.7–1.5	−242.5
Patient 11	10 / M / 36	MDZ	N/A	No response	5	1–1.5	−174.3
Patient 12	11 / M / 33	Propofol window	No Effect	No response	5	1–1.5	−221.5
Patient 13	13 / M / 81	Propofol window	No Effect	No response	5	1.6–1.8	−62.7

DZP, diazepam; LRZ, lorazepam; CNZ, clonazepam; MDZ, midazolam; GPED, generalized periodic epileptiform discharges.

a Glasgow‐Pittsburgh Cerebral Performance Categories.

b Complete resolution of GPEDs with emergence of θ and α rhythms was recorded two days later after AED increased from LEV 500bd IV to LEV 1500bd and VPA 300bd, both IV.

## Results

A total of 508, 678, and 589 GPED events were obtained for groups 1, 2, and 3 respectively.

### Voltage scalp maps

**Figure 1:**
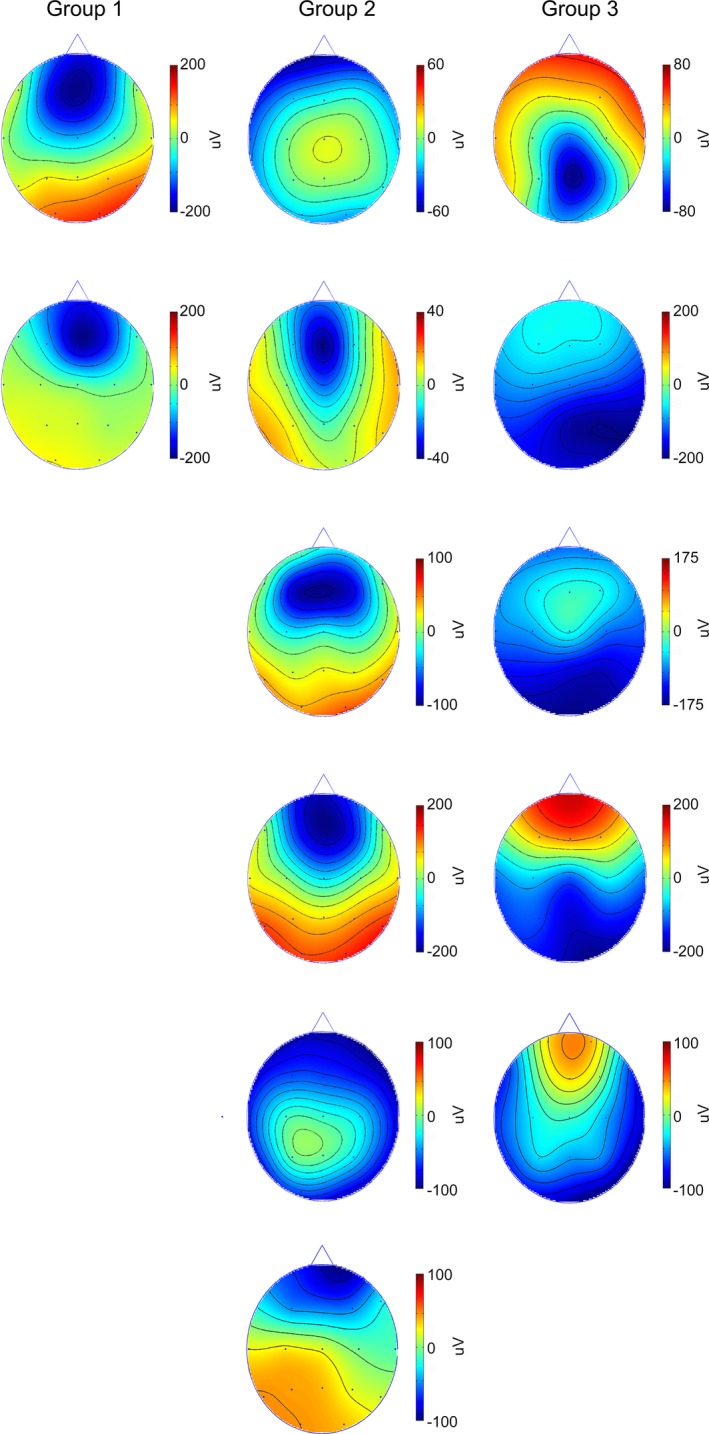
Voltage maps of the maximum negative phase of the averaged generalized periodic epileptiform discharges (GPEDs). For the possible nonconvulsive status epilepticus–related groups 1 and 2, the negative phase of the GPEDs is maximal over the frontal areas, whereas for the patients with anoxic damage (group 3), it is maximal over the posterior areas.

The topography of the negative phase of the GPEDs differed between the patients with NCSE (group 1) and those with BA (group 3). In all patients of group 1, the maximum peak of the negative GPED phase was localized over the prefrontal and superior frontal areas (Fig. 1, left panel), whereas in all patients with BA it was localized over the parietal and occipital areas (Fig. 1, right panel). All patients of group 2 (with possible NCSE) showed voltage map topographies similar to those with NCSE (Fig. 1, middle panel).

### GPED morphology

Morphology, amplitude, and duration of the GPED complexes varied inconsistently among patients within each group and across the three groups without characteristic features that could differentiate between NCSE and BA (Table 1, Fig. S2).

## Discussion

Nonevolving GPEDs are highly associated with NCSE,^10^ but they may also be “nonepileptic” as in metabolic and other encephalopathies. In postanoxic coma after CA, slow nonevolving GPEDs may still signal possible NCSE contributing to coma, or be “nonepileptic,” reflecting terminal anoxic damage.

We herein provide neurophysiological evidence, according to which an anterior localization of the maximum GPED negativity is a possible distinctive biomarker for NCSE‐related GPEDs, differentiating them from “nonepileptic” GPEDs of comparable morphology but posterior location of the major negative phase. The reason for such anterior emphasis for the “epileptic” GPEDs in postanoxic coma is unknown; perhaps one could draw a parallel with the also frontally dominant EEG discharges in generalized epilepsies and their relation to arousal mechanisms,^11^ allowing a tentative hypothesis of still intact anterior corticothalamic circuits that are responsible for arousal mechanisms.

We emphasize that anterior topography of maximal negativity of slow postanoxic GPEDs may only indicate the possibility of NCSE, but not the degree to which the latter may contribute to the coma. Moreover, its usefulness at the individual patient level, as shown in this report, has to be confirmed in a larger number of carefully studied patients. Notwithstanding these limitations, this preliminary evidence suggests that studying the EEG microstructure by voltage mapping and possibly other computational methods may reveal important biomarkers to assist the classical clinical and EEG diagnosis of NCSE.

## Disclosure

The authors declare no conflicts of interest. We confirm that we have read the Journal's position on issues involved in ethical publication and affirm that this report is consistent with those guidelines.

## Supporting information


**Figure S1.** Patient 8 (group 2). (**A**) A 2‐Hz generalized periodic epileptiform discharge (GPEDs) pattern on sedation with propofol. (**B**) Temporary discontinuation of propofol resulted in the brief frontal seizure discharges interspersing the GPEDs pattern.

**Figure S2.** Generalized periodic epileptiform discharge (GPEDs) morphology. Time‐average and superposition plots for the prominent GPEDs wave of each patient are shown.

**Appendix S1.** EEG recording and setup.
