# Supplementary figures and images for: Topography of generalized periodic epileptiform discharges in postanoxic nonconvulsive status epilepticus
Source: Epilepsia Open. 2017 Aug 21;2(4):472–5. doi: 10.1002/epi4.12073 (PMC5862105; doi:10.1002/epi4.12073)

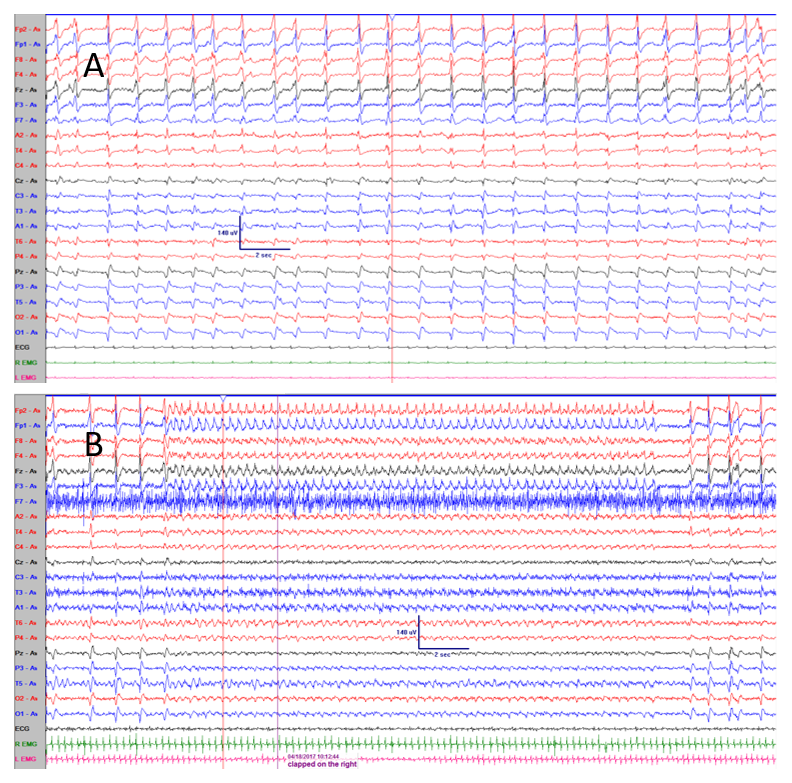

Supplement: Supplementary file 1 — Figure S1. Patient 8 (group 2). (A) A 2‐Hz generalized periodic epileptiform discharge (GPEDs) pattern on sedation with propofol. (B) Temporary discontinuation of propofol resulted in the brief frontal seizure discharges interspersing the GPEDs pattern. [file EPI4-2-472-s001.tif]

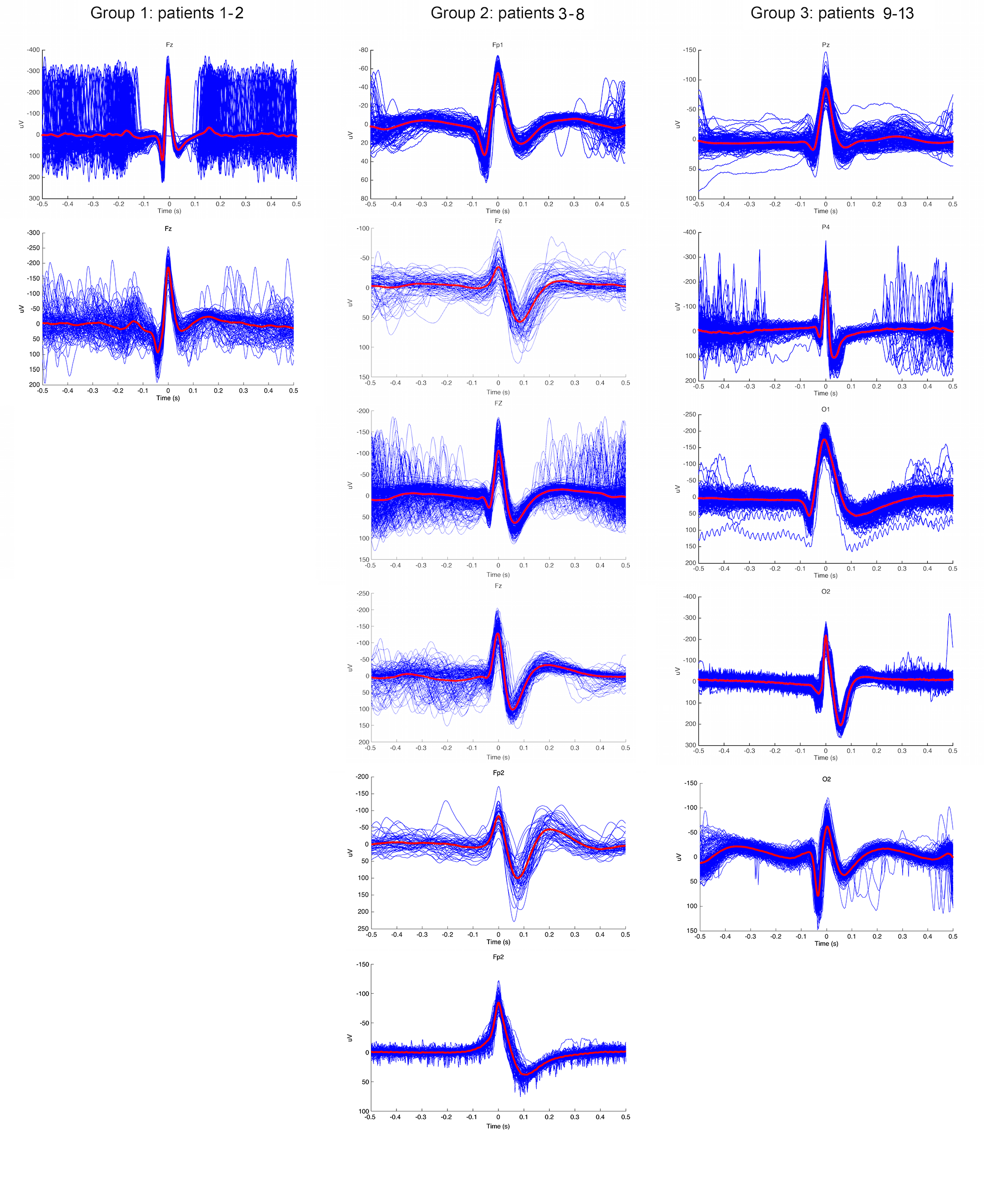

Supplement: Supplementary file 2 — Figure S2. Generalized periodic epileptiform discharge (GPEDs) morphology. Time‐average and superposition plots for the prominent GPEDs wave of each patient are shown. [file EPI4-2-472-s002.tif]
