# Supplementary material for: Topography of generalized periodic epileptiform discharges in postanoxic nonconvulsive status epilepticus
Source: Epilepsia Open. 2017 Aug 21;2(4):472–5. doi: 10.1002/epi4.12073 (PMC5862105; doi:10.1002/epi4.12073)
Supplement: Supplementary file 3 — Appendix S1. EEG recording and setup. [file EPI4-2-472-s003.docx]

Appendix (Supplementary material) EEG recording and setup.

All EEGs were recorded according to the 10-20 international system. The reference used was derived from linked C3 and C4 electrode positions and grounded over the Fz position. Impedance of all electrodes was kept below 10kOhms for most of the recording time. All electrophysiological parameters were AC recorded, amplified at a total gain of 1000, band-pass filtered at 0.05-500Hz and digitized through an 16-bit resolution A/D converter which provided accuracy of 0.084 μV/LSB, at a sampling frequency of 500Hz by a Nihon-Kohden system and stored on hard disk drives.

|  | **Patients/sex/age** | **Reactivity** | | | **Outcome*** | **GPEDs frequency (Hz)** | **GPEDs Amplitude (uV)** |
| --- | --- | --- | --- | --- | --- | --- | --- |
|  |  | **Anti-seizure manoeuver** | **EEG response** | **Clinical Response** |  |  |  |
| Patient 1 | 1 / F / 52 | None | N/A | N/A | 3 | 2.5-3.0 | -275.6 |
| Patient 2 | 2 / M / 55 | DPZ | GPEDs ceased; θ, α and β | Opened eyes to command | 2 | 2.5-3.0 | -187 |
| **Group 2** |  |  |  |  |  |  |  |
| Patient 3 | 3 / M / 63 | LRZ | GPEDs ceased; δ, θ, α and β | No | 3 | 1.5-2 | -55.69 |
| Patient 4 | 4 / M / 62 | CNZ | GPEDs ceased; δ and θ | No | 3 | 0.8 - 1.5 | -34.8 |
| Patient 5 | 5 / M / 60 | Propofol window | GPEDs ceased; δ and θ, some α | No | 5 | 1.0 | -110.7 |
| Patient 6 | 6 / M / 60 | None | N/A** | No response | 3 | 1.2-2.0 | -83 |
| Patient 7 | 7 / M / 71 | DZP | GPEDs ceased; δ and θ, some α | No | 3 | 1.2 - 1.8 | -130.6 |
| Patient 8 | 8 / M / 70 | Propofol window | No physiological rhythms, but focal frontal seizure activity | No | 5 | 0.8 - 1.2 | -74.8 |
| **Group 3** |  |  |  |  |  |  |  |
| Patient 9 | 8 / M / 58 | LZP | No Effect | No response | 3 | 1.5-2.0 | -86.4 |
| Patient 10 | 9 / M / 78 | LZP | No Effect | No response | 5 | 0.7-1.5 | -242.5 |
| Patient 11 | 10 / M / 36 | MDZ | N/A | No response | 5 | 1-1.5 | -174.3 |
| Patient 12 | 11 / M / 33 | Propofol window | No Effect | No response | 5 | 1-1.5 | -221.5 |
| Patient 13 | 13 / M / 81 | Propofol window | No Effect | No response | 5 | 1.6-1.8 | -62.7 |
|  |  |  |  |  |  |  |  |
| * Glasgow-Pittsburgh Cerebral Performance Categories | | |  |  |  |  |  |
| **complete resolution of GPEDs with emergence of θ and α rhythms was recorded two days later after AED increased from LEV 500bd IV to LEV 1500bd and VPA 300bd, both IV | | | | | | | |
